# Short-interval second ejaculation improves sperm quality, blastocyst formation in oligoasthenozoospermic males in ICSI cycles: a time-lapse sibling oocytes study

**DOI:** 10.3389/fendo.2023.1250663

**Published:** 2023-09-07

**Authors:** Yaoxuan Li, Shikai Wang, Dawen Li, Yueyue Huang, Haifang Liu, Xiaohui Zhang, Jie Qin, Xianbao Mao, Zhengda Li, Liangshi Chen, Pingpin Wei, Wen Shi, Lintao Xue

**Affiliations:** Reproductive Medical and Genetic Center, The People’s Hospital of GuangXi Zhuang Autonomous Region, Nanning, China

**Keywords:** abstinence period, sperm quality, embryo development, sibling oocyte, time lapse

## Abstract

**Background:**

Does short-interval second ejaculation improve sperm quality, embryo development and clinical outcomes for oligoasthenozoospermia males received intracytoplasmic sperm injection (ICSI) treatment?

**Methods:**

All enrolled male patients underwent short-interval secondary ejaculation on the day of oocyte retrieval, and 786 sibling MII oocytes from 67 cycles were equally divided into two groups based on whether the injected spermatozoons originated from the first or second ejaculation. Semen parameters, embryo development efficiency, morphokinetic parameters and clinical outcomes were compared between the two groups to assess the efficiency and clinical value of short-interval second ejaculation in ICSI cycles.

**Results:**

Short-interval second ejaculation significantly improved sperm motility, normal morphological rate, and sperm DNA integrity both before and after sperm swim-up. The high-quality blastocyst rate (24.79% versus 14.67%), available blastocyst rate (57.56% versus 48.44%), and oocyte utilization rate (52.93% versus 45.29%) were significantly higher in the second ejaculation group (*P*<0.05). The clinical pregnancy rate (59.09% versus 47.37%), implantation rate (42.11% versus 32.35%) and live birth rate (40.91% versus 31.58%) were higher in the second ejaculation group, but the differences were not significant (*P*>0.05). Time-lapse analysis showed that morphokinetic time points after the 7-cell stage were earlier in the second ejaculation group but without a significant difference (*P*>0.05), and abnormal embryo cleavage patterns between the two groups were not significantly different (*P*>0.05).

**Conclusions:**

Short-interval second ejaculation significantly improves sperm quality in oligoasthenozoospermic males, and is beneficial for blastocyst formation efficiency in ICSI cycles. This study suggested a non-invasive and simple but effective strategy for improving ICSI treatment outcomes.

## 1 Introduction

In the past few decades, the global incidence of infertility has been increasing year by year, with male factors accounting for approximately 40-50% among infertile couples (1). Additionally, several studies have indicated a significant decline in sperm quality over the past two decades (2, 3). As the most widely used assisted reproductive technology (ART) procedure, ICSI is the most effective clinical treatment for male-factor infertility caused by poor sperm quality. However, patients with poor sperm motility not only exhibit abnormalities in conventional parameters but also typically present with severe functional defects that include high levels of sperm DNA fragmentation and abnormal sperm chromatin packaging (4, 5). Sperm abnormalities significantly affect ICSI fertilization rate, embryo development efficiency and clinical treatment outcome (6). Therefore, improving sperm quality in ICSI patients would be beneficial in enhancing clinical treatment efficacy.

Oligoasthenozoospermia accounts for approximately half of male infertility cases and is the main cause of male infertility, with the age of onset becoming increasingly younger (7, 8). Oligoasthenozoospermia is characterized primarily by low sperm concentration or motility. Previous studies have shown that patients with low sperm motility have slightly higher levels of ROS and sperm DNA fragmentation than normal males (9, 10). Elevated levels of ROS in semen can result in oxidative stress, causing damage to sperm DNA, sperm plasma membrane, and mitochondrial function (11).

Sperm are generated in the seminiferous tubules of the testes, migrate to the epididymis for maturation and are stored in the tail of the epididymis until ejaculation (12). As the duration of sperm retention in the epididymis and vas deferens increases, alterations in the semen environment along with the build-up of ROS may lead to sperm damage (13, 14). The recom-mended abstinence period (2-7 days) by the World Health Organization (WHO) is only for semen analysis, and not a reference standard for male ejaculation abstinence time in ART treatment, but most laboratories still choose semen with an abstinence time of 2-7 days for fertilization. It has been thoroughly demonstrated that the abstinence duration influences sperm parameters. Long-term abstinence can result in semen volume and sperm concentration, while shorter abstinence periods mean higher sperm motility and lower sperm DNA fragmentation rates, and lower DNA fragmentation rates favor improved male fertility potential (14–16).

Meanwhile, there are few studies on the impact of abstinence time on the clinical outcomes of IVF/ICSI. Some research has showed that shorter abstinence periods can improve fertilization rates, blastocyst formation rates, and clinical outcomes (17–19). Conversely, a few studies showed that it did not lead to an obvious improvement in the fertilization rates, embryo quality, pregnancy rate or live birth rates (20, 21). Thus, the conclusions regarding the effect of abstinence duration on clinical outcomes are still controversial. However, most of the current studies could not strictly control for confounding factors such as female age, ovarian reserve function, and superovulation protocols, which led to inconsistent conclusions. Therefore, sibling oocyte studies could eliminate the bias caused by female factors and more clearly reflect the impact of different sperm sources on clinical outcomes. Additionally, time-lapse imaging could be used to analyse the morphokinetic behaviour of embryos based on early morphokinetic parameters, which is beneficial for studying the impact of sperm factors on embryonic development, but there is currently no relevant research.

This study designed a time-lapse sibling oocyte trial based on oligoasthenozoospermic males who received ICSI treatment. First, we analysed semen samples collected from the same male after short-interval consecutive ejaculations. Then, sibling MII oocytes were randomly assigned to the first or second ejaculation group, fertilization and embryo development were observed by time-lapse imaging, and clinical outcomes after embryo transfer were analysed. The purpose of the present study was to indicate whether short-interval second ejaculation could improve sperm quality and clinical outcomes in ICSI cycles.

## 2 Methods

### 2.1 Patient population, ovarian stimulation and oocyte retrieval

This study recruited patients who received ICSI treatment in the period between March 2021 to March 2023 at the People’s Hospital of Guangxi Zhuang Autonomous Region. All enrolled patients were required to undergo short-interval secondary masturbation and semen collection on the day of oocyte retrieval, and the inclusion criteria were as follows: (1) semen could be collected by masturbation on the day of oocyte retrieval; (2) sperm concentration<5×10^6^ or progressive motility rate<10% before sperm preparation; and (3) total number of progressive motile sperm< 1×10^6^ after sperm preparation. Patients with the following conditions were excluded: (1) failure to obtain a second ejaculation sample from the male partner; and (2) number of MII oocytes available for ICSI less than two. All enrolled patients signed informed consent and the study was approved by the Ethics Committee of the People’s Hospital of Guangxi Zhuang Autonomous Region at 25 February 2021(Approval No: LL-KY-ZC-2021-02). Ovarian stimulation and oocytes retrieval were performed by the stander protocol as described previously (22).

### 2.2 Oocyte allocation and ICSI procedure

The cumulus cells surrounding the retrieved oocytes were removed mechanically at 80 IU/ml hyaluronidase (Irvine Scientific, California, USA) preheated to 37°C, all MII oocytes were confirmed by one embryologist using an inverted microscope and then transferred into a droplet containing 30 μl of G-IVF PLUS™ (10136, Vitrolife) medium covered with mineral oil (Vitrolife, Goteborg, Sweden).Then, another embryologist randomly allocated the MII oocytes into two groups: the first ejaculation group, in which MII oocytes were injected with spermatozoons derived from the first ejaculation after a long abstinence period, and the second ejaculation group, in which MII oocytes were injected with spermatozoons derived from a short-interval second ejaculation. The injection procedure was performed by the same embryologist. The injected MII oocytes were transferred into G-1 PLUS™ (10128, Vitrolife), covered with mineral oil in a Primo Vision dish (9-well or 16-well, Vitrolife, Viby, Denmark) and cultured in a Primo Vision (Vitrolife, Budapest, Hungary) incubator at 37°C, 6% CO_2_, and 5% O_2_.

### 2.3 Semen collection and quality analysis

After abstinence for 2-10 days, all male patients provided semen through the first ejaculation by masturbation on the oocyte retrieval day, and then performed second ejaculation after a short-interval(≤3h) of rest. Semen quality analysis was carried out according to the fifth edition of the WHO guidelines. Semen volume was measured using the weighing method. After liquefaction, the SCA^®^ sperm quality analysis system (MicroPtic, Spain) was used for concentration and motility analysis. Diff-Quik staining was used for sperm morphology analysis. After smear staining, sperm morphology was analysed under an oil immersion lens at 1000× magnification. At least 200 sperm were analysed per field. The SCD test was used to detect the sperm DNA fragmentation rate, and the operation was carried out according to the instructions of the halosperm^®^ kit (halotech^®^, Spain). The criteria for determining DNA fragmentation sperm were that the width of the halo around the sperm head was less than one-third of the diameter of the sperm nucleus, and the sperm head had no halo or was not stained. The proportion of sperm with these features in the total observed sperm was the sperm DNA fragmentation rate, and at least 500 spermatozoa were analysed per sample.

### 2.4 Fertilization check, embryo assessment and time-lapse monitoring

On the first day after ICSI injection, the fertilization status was evaluated by observing pronuclei in the cytoplasm. Two pronuclei observed indicated normal fertilization. On the third day, the quality of cleavage stage embryos was evaluated based on factors such as cleavage speed, uniformity of blastomeres, and fragmentation rate. According to the Istanbul consensus (Alpha Scientists in Reproductive Medicine (23), embryos derived from normally fertilized oocytes with 7-9 cells and less than 10% fragmentation on the third day were defined as high-quality embryos. On the fifth and sixth day, the quality of blastocysts was evaluated using the Gardner scoring system (24). The blastocysts were divided into 6 stages based on the size of the blastocoel and degree of hatching. Blastocysts at or beyond stage 3 were classified into three grades (A, B, C) based on the number of inner cell mass and trophectoderm cells. Blastocysts 4BB and above were defined as high-quality blastocysts, and blastocysts that were better than 3BC or 3CB were defined as available blastocysts.

Embryos are cultured and monitored in a time-lapse monitoring incubator (37°C, 6% CO2, 5% O2) connected to the Analyzer image analysis software of Primo Vision (Primo Vision Evo, Vitrolife, Hungary), while their developmental process is recorded. According to the proposed guidelines for the annotation of dynamic human embryo monitoring (25), the parameters for dynamic time points include: the second polar body is completely detached from the oolemma(tPB2); time of appearance of pronuclei(tPNa); time of pronuclei disappearance (tPNf); two to nine discrete cells (t2 to t9); initiation of blastulation (tSB); expansion blastocyst (tB); initiation of hatching process(tHN); t3-t2(CC1); t4-t3(S2); tPNf-t5 (t5-tPNf) and t8-t5(S3). There are 5 types of abnormal cleavage of embryos: The embryo divides directly from one cell to≥3 cells before 8 cells (DC). Two separate cells recombine into one cell before 8 cells (RC), During the first division, cells divide irregularly into more than 4 cells, accompanied by the production of a large number of fragments (CC), Multi-pronuclei in any one cell at the 2-cell or 4-cell stage (MN) and there are two or more cleavage patterns in one embryo (MIX). All dynamic developmental parameters and abnormal cleavage patterns were reviewed and confirmed by two experienced embryologists through double-checking.

### 2.5 Embryo transfer

No more than two embryos were transferred in any transfer cycle. The embryos of a single fresh transfer cycle were all derived from the same sperm source group, depending on which group the best quality embryos came from. On day 3 or day 5, embryos were transferred into the uterine cavity.

### 2.6 Outcomes

The oocyte utilization rate was the primary outcome of our research. The secondary outcomes were the blastocyst formation rate, high-quality blastocyst rate, clinical pregnancy rate, embryo implantation rate and early miscarriage rate. Furthermore, we evaluated the fertilization and day3 embryo development outcomes, embryo morphokinetic parameters, sperm quality parameters from different groups.

The oocyte utilization rate was calculated as the total number of embryos transferred or frozen derived from a single oocyte retrieval cycle divided by the number of total injected MII oocytes. Blastocyst formation rate was calculated as blastocysts number divided by the total number of day3 extended cultured embryos. High-quality blastocysts rate was calculated as high-quality blastocysts number divided by the total number of day3 extended cultured embryos. The biochemical pregnancy rate was defined as serum hCG > 10mIU/ml 14 days after transplantation. Clinical pregnancy was defined as the presence of an intrauterine gestational sac, and heartbeat was confirmed by ultrasound sound examination at the 4th week after embryo transfer. Early miscarriage was defined as pregnancy loss before 12 weeks. Ongoing pregnancy was defined as an intact pregnancy without termination of the pregnancy before the 12th week of gestation. Live birth was defined as any birth event in which at least one baby was born alive and survived for more than 1 month.

### 2.7 Statistical analysis

Statistical analysis was carried out using SPSS Statistics 26.0 software (IBM SPSS Corp., USA). Quantitative data were tested by Kolmogorov-Smirnov test to check whether the data conformed to the normal distribution at first. Quantitative data conforming to the normal distribution were presented by the mean ± standard deviation ( $\bar{x}$ ± s), and those that are not normal distribution were presented as the median [quartile1 (Q1), quartile 3, (Q3)], and independent-sample *t-*test or Mann-Whitney nonparametric tests were used to compare the mean values as appropriate. the categorical data are presented as percentages (%), while the chi-square test was used to compare the rates of two groups. A *P* value< 0.05 indicated that the difference was statistically significant.

## 3 Results

**Figure 1:**
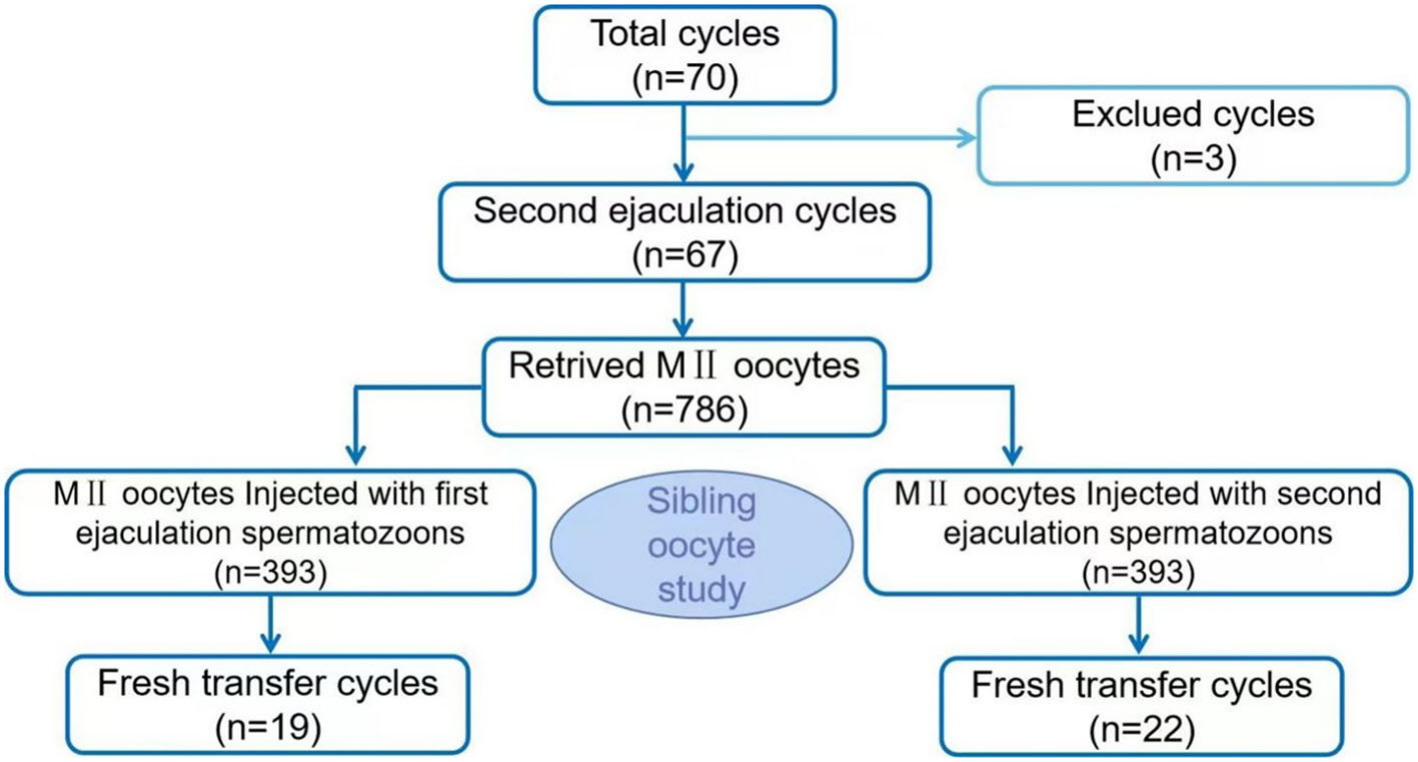
Flow chart of the study design.

Sixty-seven patients were ultimately included in the study, while 3 patients were excluded (1 patient refused and 2 patients failed to secondary ejaculation attempts). The success rate of secondary ejaculation was 95.71% (67/70). Data from 67 patients were analysed. The average age of females was 32.85 ± 3.88 years, and the average age of males was 34.52 ± 5.34 years. A total of 927 oocyte cumulus corona complexes (OCCCs) were retrieved, with an average of 13.84 ± 5.19 oocytes per cycle, and 786 MII oocytes were divided equally into two groups for injection with spermatozoon obtained through the first ejaculation or second ejaculation respectively (**Figure 1**). In general, the total 2PN fertilization rate, D3 high-quality embryo rate, high-quality blastocyst rate, available blastocyst rate and oocyte utilization rate were 76.97%, 68.93%, 24.79%, 57.56%, and 49.11%, respectively. The baseline characteristics and embryonic parameters of the 67 patients are shown in **Table 1**.

**Table 1:** Characteristics and basic embryo data of the enrolled patients.

Total cycles(n)	70
Successful secondary ejaculation cycles(n)	67
Short-term second ejaculation success rate (%)	95.71 (67/70)
Female age, years (mean ± SD)	32.85 ± 3.88
Infertility duration, years (mean ± SD)	3.63 ± 2.13
Female BMI (mean ± SD)	21.80 ± 3.18
FSH (mean ± SD)	6.91 ± 1.74
Male age, years (mean ± SD)	34.52 ± 5.27
Male BMI (mean ± SD)	24.62 ± 4.88
Average No. Of oocytes retrieved (mean ± SD)	13.84 ± 5.19
MII oocyte rate (%)	84.79 (786/927)
2PN fertilization rate (%)	76.97 (605/786)
2PN cleavage rate (%)	99.67 (603/605)
Day3 high-quality embryos rate (%)	68.93 (417/605)
Blastocyst formation rate (%)	69.11 (320/463)
High-quality blastocyst rate (%)	24.79 (92/463)
Available blastocyst rate (%)	57.56 (246/463)
Oocyte utilization rate (%)	49.11 (386/786)

Comparison of semen parameters before and after the sperm swim-up method between the first ejaculation group and the second ejaculation group (**Table 2**) clearly revealed the significant effect of the length of abstinence time on semen quality. Compared to the first ejaculation, the semen volume of the second ejaculation decreased significantly (*P*<0.001), but the decrease in semen volume did not lead to a reduction in sperm concentration (*P*>0.05). The total motility, progressive motility and normal morphological sperm rate in the Second ejaculation group were significantly higher than those in the first ejaculation group both before and after swim-up(*P*<0.05). On the other hand, compared with that of the first ejaculation group, the sperm DNA fragmentation of the second ejaculation group significantly decreased both before and after swim-up (*P*<0.05).

**Table 2:** Comparison of semen parameters in 72 patients stratified by sperm swim-up technique.

Parameters	First ejaculation	Second ejaculation	Z-*value*		*P-value*
Before swim-up					
Abstinence period	4 days (3, 4)	85min (75, 95)	/		*/*
Seminal volume (ml)	2.50 (1.50, 3.00)	1.50 (0.80, 2.00)	-5.871		<0.001
Sperm concentration (×10^6/^ml)	12.80 (6.00, 22.20)	10.1 (4.90, 23.40)	-0.708		0.479
progressive motility (%)	5.80 (3.60, 13.00)	11.8 (4.70, 19.00)	-2.942		0.003
Total motility (%)	22.70 (15.70, 33.90)	32.0 (20.90, 43.20)	-2.570		0.010
Normal morphological sperm rate (%)	1.00 (1.00, 2.00)	2.00 (1.00,3.00)	-2.263		0.024
Sperm DNA fragmentation (%)	35.00 (24.0, 46.00)	28.00 (15.00,39.00)	-2.424		0.015
After swim-up					
Seminal volume (ml)	0.50	0.50	/		/
Sperm concentration (×10^6/^ml)	0.90 (0.50,1.80)	1.00 (0.70,1.30)	-0.499		0.618
progressive motility (%)	63.20 (45.80, 70.20)	70.80 (56.00,80.00)	-2.924		0.003
Total motility(%)	87.50 (68.80, 96.70)	90.00 (85.00,97.40)	-1.763		0.078
Normal morphological sperm rate(%)	1.00 (1.00,2.00)	2.00 (1.00, 3.00)	-2.148		0.032
Sperm DNA fragmentation (%)	8.00 (5.00,11.00)	5.00 (4.00, 8.00)	-0.357		<0.001

The comparison of embryonic development and clinical outcomes between the two groups is presented in **Table 3**. There were no significant differences in the 2PN fertilization rate (75.83% versus 78.12%), day-3 high-quality embryo rate (68.79% versus 69.06%), or blastocyst formation rate (66.67% versus 71.43%) between the two groups(*P*>0.05). However, the high-quality blastocyst rate (24.79% versus 14.67%), available blastocyst rate (57.56% versus 48.44%), and oocyte utilization rate (52.93% versus 45.29%) were significantly higher in the second ejaculation group than in the first ejaculation group (*P*<0.05).

**Table 3:** Comparison of laboratory, clinical outcomes, fertilization and embryo development between two groups.

	First ejaculation	Second ejaculation	Z/χ^2^ *value*	*P-value*
No. Of injectable MII oocytes	393	393		
Oocyte degradation rate (%)	2.54 (10/393)	3.31 (13/393)	0.403	0.525
2PN fertilization rate (%)	75.83 (298/393)	78.12 (307/393)	0.581	0.446
2PN cleavage rate (%)	100.00 (298/298)	99.35 (305/307)	1.948	0.163
Day3 high-quality embryos rate (%)	68.79 (205/298)	69.06 (212/307)	0.005	0.944
Blastocyst formation rate (%)	66.67 (150/225)	71.43 (170/238)	1.229	0.268
High-quality blastocyst rate (%)	14.67 (33/225)	24.79 (59/238)	7.444	0.006
Available blastocyst rate (%)	48.44 (109/225)	57.56 (137/238)	3.862	0.049
Oocyte utilization rate (%)	45.29 (178/393)	52.93 (208/393)	4.582	0.032
Embryo transfer cycles(n)	19	22		
Female age, years [M (P25, P75)]	32.00 (30.00, 35.00)	33.50 (29.00, 36.00)	-0.013	0.990
Male age, years [M (P25, P75)]	34.00 (29.00, 37.00)	33.50 (30.00,37.50)	-0.066	0.948
No. Of embryo transferred (n)	34	38		
Average no. Of embryo transferred, n [M (P25, P75)]	2.00 (2.00, 2.00)	2.00 (1.00, 2.00)	-0.457	0.648
Biochemical pregnancy rate (%)	57.89 (11/19)	63.64 (14/22)	0.141	0.707
Clinical pregnancy rate (%)	47.37 (9/19)	59.09 (13/22)	0.563	0.453
Implantation rate (%)	32.35 (11/34)	42.11 (16/38)	0.728	0.393
Early miscarriage rate (%)	5.26 (1/19)	4.55(1/22)	0.011	0.915
Ongoing pregnancy rate (%)	10.53 (2/19)	13.64(3/22)	0.092	0.722
Live birth rate (%)	31.58 (6/19)	40.91(9/22)	0.383	0.536

A total of 41 fresh embryo transfer cycles were performed. The first ejaculation group and second ejaculation group included 19 and 22 cycles, respectively. However, there was no significant difference (*P*>0.05) in the average number of embryos transferred (1.79 ± 0.42 versus 1.73 ± 0.46). The biochemical pregnancy rate (63.64% versus 57.89%), clinical pregnancy rate (59.09% versus 47.37%), implantation rate (42.11% versus 32.35%) and live birth rate (40.91% versus 31.58%) in the second ejaculation group were higher than those in the first ejaculation group, and the second ejaculation group had a lower early miscarriage rate (4.55% versus 5.26%). However, there was no significant difference (*P*>0.05).

The embryo morphokinetic parameters and abnormal cleavage patterns of embryo division in the two groups are shown in **Table 4**. We observed that in the first ejaculation group, all recorded time points prior to the 7-cell stage were earlier than those in the second ejaculation group. However, from the 7-cell stage onwards, this phenomenon was reversed: the second ejaculation group had earlier recorded time points for embryonic development (t7C, t8C, tSB, tB, tHN). However, there were no significant differences in the time parameters or rate of abnormal cleavage patterns between the two groups (*P*>0.05).

**Table 4:** Comparison of embryo morphokinetic parameters and embryo cleavage patterns between two groups under time-lapse.

	First ejaculation	Second ejaculation	Z/*x* ^2^ *value*	*P-value*
Time parameters (h)				
tPB2	2.80 (2.31, 3.45)	2.90 (2.40, 3.60)	-1.780	0.075
tPNa	6.27 (5.62, 7.12)	6.40 (5.60, 7.20)	-0.958	0.338
tPNf	23.77 (21.88, 25.94)	23.70 (21.90, 26.25)	-0.045	0.964
2C	26.01 (24.25, 28.13)	26.10 (24.20, 28.38)	-0.335	0.738
3C	36.47 (33.45, 39.17)	36.20 (33.55, 39.25)	-0.185	0.853
4C	37.50 (35.05, 39.98)	37.80 (35.00, 41.05)	-1.271	0.204
5C	49.13 (43.73,53.63)	49.70 (45.30, 53.88)	-1.032	0.302
6C	51.69 (48.09, 56.53)	52.40 (48.10, 56.80)	-0.498	0.618
7C	55.12 (50.26, 60.27)	54.50 (50.40, 60.20)	-0.484	0.628
8C	57.09 (52.67, 65.52)	57.00 (51.80,64.80)	-1.049	0.294
tPNf-t5	26.60 (18.55, 29.25)	26.30 (22.72, 29.10)	-0.378	0.705
CC1	11.17 (10.33, 12.33)	11.20 (10.20, 12.20)	-0.030	0.976
S2	0.83 (0.33, 8.28)	0.90 (0.30, 3.75)	-0.027	0.978
S3	9.41 (3.73, 18.39)	6.50 (3.30, 16.80)	-1.699	0.089
tSB	102.65 (95.84,111.30)	101.90 (95.85,107.93)	-0.903	0.366
tB	114.12 (105.77,122.24)	112.60 (106.68,120.10)	-0.679	0.497
tHN	115.62 (111.71,130.80)	115.30 (112.02,129.68)	-0.262	0.793
Abnormal cleavage pattern				
Abnormal cleavage rate (%)	33.22 (99/298)	32.13 (98/305)	0.081	0.775
DC (%)	14.09 (42/298)	13.77 (42/305)	0.013	0.909
RC (%)	2.01 (6/298)	4.59 (14/305)	3.121	0.077
CC (%)	1.34 (4/298)	0.98 (3/305)	0.169	0.681
FC (%)	2.01 (6/298)	1.97 (6/305)	0.002	0.968
MN (%)	7.38 (22/298)	4.92 (15/305)	1.590	0.207
MIX (%)	6.38 (19/298)	5.90 (18/305)	0.059	0.808

## 4 Discussion

To our knowledge, this is the first time-lapse sibling oocyte study to explore the impact of short-interval second ejaculation on sperm quality, embryo development and clinical outcomes. It is widely recognized that female factors, especially oocyte quality, are the main factors interfering with the influence of sperm quality on embryo development. The study design of sibling oocytes could minimize the bias caused by female factors, thus leading to the most reliable conclusion regarding the clinical application value of short-interval second ejaculation. This study demonstrated that short-interval second ejaculation not only significantly improved sperm quality but was also beneficial for embryonic development, in particular for blastocyst formation efficiency.

There are great individual differences in semen characteristics, but the period of abstinence is a certain factor affecting the characteristics of subjects (12). Many studies have shown that a short abstinence period might improve the morphology, motility, and other semen parameters (26–29). Our study found that short-term abstinence can cause a decrease in semen volume but signifi-cantly improve sperm motility, the normal morphological rate and DNA integrity. An interesting study showed that there are differentially expressed proteins in seminal plasma during different periods of abstinence that are highly involved in the improvement of fertilization ability and sperm motility in males with a short abstinence period (30). In addition, with long ejaculatory abstinence, senescent spermatozoa progressively accumulate, and produce excessive ROS, which damages sperm motility and sperm membrane integrity, and on the other hand, it directly leads to increased DNA fragmentation (14, 31). Our results further strengthen the notion that short-term abstinence benefits sperm quality.

Achieving a live birth is considered the ultimate goal of ART, so further research is necessary to determine whether short-interval second ejaculation has positive implications for both embryo development and clinical outcomes. Scarselli’s study discovered that using sperm collected with an extremely short abstinence period in ART can improve embryo development and significantly decrease the rate of aneuploid blastocysts (17). However, some studies have found that shortening the abstinence time did not affect embryonic development (20, 21). Specifically, the results of this study demonstrate that short-term secondary ejaculation could improve embryo development. The conclusion is drawn from the significantly higher rates of high-quality blastocysts (14.67% versus 24.79%, *P*=0.006) and available blastocysts (48.44%versus57.56%, *P*=0.049) in the second ejaculation group that in the first ejaculation group. Additionally, the oocyte utilization rate (45.29% versus 52.96%, *P*=0.032) was significantly elevated in the second ejaculation group, suggesting an increased chance for couples to achieve successful offspring. Moreover, under time-lapse monitoring, an interesting finding was that embryos in the second ejaculation group cleaved into 2-6 cells later than those in the first ejaculation group. However, after 7 cells, this phenomenon was reversed: the time parameters of embryo and blastocyst development in the second ejaculation group were earlier.

We speculate that there may be a correlation between the promotion of sperm quality, especially the enhancement of sperm DNA integrity, and the improvement of blastocyst quality. Several recent studies have found that the formation of blastocysts is closely related to the high condensation of paternal and maternal chromosomes. An increase in the sperm DNA fragmentation rate can affect the compact state of paternal chromosomes, leading to a decrease in the fertilization rate and blastocyst formation rate after ICSI (32–34). However, the paternal genome also affects the developmental speed of the embryo. As stated in a recent study, poor DNA integrity in sperm can cause delayed embryo development, which may be related to the extended time required for oocytes to repair paternal genetic material (33). Furthermore, from a more microscopic perspective of molecular biology, after the maternal genome controls are transformed during the 8-16 cell stage of human embryos, transcriptional activation of the paternal genome begins to participate in regulating the process of embryonic development (35, 36). This may also be one of the reasons why the late embryo and blastocyst development time point in the second ejaculation group was earlier in this study.

Notably, a compelling systematic review suggests that the current evidence indicates that shorter abstinence periods lead to better clinical pregnancy and live birth rates (37). Furthermore, a recent systematic review and meta-analysis performed to compare very short abstinence periods on ART outcomes found that the implantation rate, clinical pregnancy rate, and live birth rate among these treatment-seeking couples improved significantly (38). We found that a short-interval second ejaculation could lead to a higher clinical pregnancy rate, implantation rate and live birth rate, but there were no significant differences due to the limited sample size. On the other hand, it is worth mentioning that the oocyte utilization rate was significantly higher in the second ejaculation group. This suggested that patients had access to more viable embryos during a single retrieval oocyte cycle, thereby enhancing their prospects of attaining pregnancy and live birth in consequent thawing cycles. The influence of short-term abstinence on clinical outcomes requires further investigation.

In conclusion, this study demonstrated that a short-interval second ejaculation can lead to higher sperm motility, a normal morphological rate and DNA integrity, and an improvement in embryonic development, especially blastocyst formation efficiency, was proven through a sibling oocyte study based on males with oligoasthenozoospermia. For males with poor sperm quality, short-interval second ejaculation is a non-invasive and simple but effective strategy to improve clinical outcomes. Although these results are promising, this study was based on oligoasthenozoospermic males who received ICSI treatment, and whether other types of infertile males or even those undergoing IVF cycles could also benefit from short-interval second ejaculation needs to be investigated further.

## Data availability statement

The raw data supporting the conclusions of this article will be made available by the authors, without undue reservation.

## Ethics statement

The studies involving humans were approved by Ethics Committee of the People’s Hospital of Guangxi Zhuang Autonomous Region. The studies were conducted in accordance with the local legislation and institutional requirements. The participants provided their written informed consent to participate in this study.

## Author contributions

YL analysed the data and drafted the manuscript. SW collected and statistically analysed the data. DL and JQ communicated with the patients and collected consent for participation. YH, LC, XZ and WS tested the semen and collected data. XM, ZL and PW conducted embryo processing and data collection. LX proofread the manuscript and made revisions. All authors contributed to the article and approved the submitted version.
